# Attentional Set-Shifting Deficit in Parkinson’s Disease Is Associated with Prefrontal Dysfunction: An FDG-PET Study

**DOI:** 10.1371/journal.pone.0038498

**Published:** 2012-06-07

**Authors:** Yoichi Sawada, Yoshiyuki Nishio, Kyoko Suzuki, Kazumi Hirayama, Atsushi Takeda, Yoshiyuki Hosokai, Toshiyuki Ishioka, Yasuto Itoyama, Shoki Takahashi, Hiroshi Fukuda, Etsuro Mori

**Affiliations:** 1 Department of Behavioral Neurology and Cognitive Neuroscience, Tohoku University Graduate School of Medicine, Sendai, Japan; 2 Department of Clinical Neuroscience, Yamagata University Graduate School of Medicine, Yamagata, Japan; 3 Department of Occupational Therapy, Yamagata Prefectural University of Health Science, Yamagata, Japan; 4 Department of Neurology, Tohoku University Graduate School of Medicine, Sendai, Japan; 5 Department of Diagnostic Radiology, Tohoku University Graduate School of Medicine, Sendai, Japan; 6 Department of Radiology and Nuclear Medicine, Institute of Development, Aging, and Cancer, Tohoku University, Sendai, Japan; University of Granada, Spain

## Abstract

The attentional set-shifting deficit that has been observed in Parkinson’s disease (PD) has long been considered neuropsychological evidence of the involvement of meso-prefrontal and prefrontal-striatal circuits in cognitive flexibility. However, recent studies have suggested that non-dopaminergic, posterior cortical pathologies may also contribute to this deficit. Although several neuroimaging studies have addressed this issue, the results of these studies were confounded by the use of tasks that required other cognitive processes in addition to set-shifting, such as rule learning and working memory. In this study, we attempted to identify the neural correlates of the attentional set-shifting deficit in PD using a compound letter task and 18F-*fluoro*-deoxy-glucose (FDG) positron emission tomography during rest. Shift cost, which is a measure of attentional set-shifting ability, was significantly correlated with hypometabolism in the right dorsolateral prefrontal cortex, including the putative human frontal eye field. Our results provide direct evidence that dysfunction in the dorsolateral prefrontal cortex makes a primary contribution to the attentional set-shifting deficit that has been observed in PD patients.

## Introduction

Cognitive inflexibility is a primary neuropsychological feature of Parkinson’s disease (PD) [1], [2]. Neuropsychological tests of ‘frontal lobe’ function, such as the Wisconsin Card Sorting Test (WCST), the Intra-Dimensional/Extra-Dimensional (ID/ED) set-shifting paradigm, the Odd-Man-Out task and variants of these tests, have been used to measure cognitive flexibility [3], [4], [5], [6]. In these tasks, subjects are shown a successive series of visual stimuli that have multiple perceptual dimensions, and they are asked to flexibly switch their behavioral responses from one particular perceptual dimension to another dimension on the basis of a pre-learned rule. The focus of interest in these tasks lies in the cognitive process involved in ‘set-shifting’, which is the process of shifting or switching between stimulus-response sets [7]. A major problem in interpreting the results of studies that use these tasks is the confounding effect of cognitive abilities other than set-shifting that are required for task performance [7], [8]. For example, performance on the WCST depends on inference and concept formation abilities, and rule-learning abilities and working memory function are major contributing factors to performance efficiency on the ID/ED paradigms and the Odd-Man-Out task. More recent studies have made substantial efforts to isolate set-shifting from these confounding factors. For instance, Cools and colleagues devised a task in which they used letters and digits instead of the abstract geometric figures that were used in the antecedent tasks [8]. Both letter and digit identification are governed by well-established stimulus-response rules, require no new learning and require little working memory, whereas the manipulation of multidimensional geometric figures demands rather high capacities for both learning and working memory. Another problem in investigating set-shifting is that there are two critical components of any given cognitive set: the stimulus set and the response set [7], [9]. Set-shifting that requires reconfiguring both the stimulus and response sets is called ‘task-set switching’, whereas set-shifting that only requires reconfiguration of the stimulus set is called ‘attentional set-shifting’. There may be differences in the mechanisms and neural bases for these distinct set-shifting processes. In the aforementioned study by Cools and colleagues, patients with PD only showed attentional set-shifting deficits when the target stimuli were presented in the company of competing stimuli [8]. Similarly, Ravizza and colleagues demonstrated that interference from competing stimuli, or stimulus ‘cross-talk,’ resulted in poorer attentional set-shifting performance on the modified Odd-Man-Out task in PD patients [10]. In contrast, a recent study by Kehagia and colleagues reported that the performances of patients with very early stages of PD (Hoehn-Yahr stages I and II) were equivalent to those of healthy control participants on a newly developed paradigm that had been designed to assess the impact of stimulus cross-talk on task-set switching performance [9]. In summary, the current evidence suggests that in situations in which competitive stimuli are present, early stage PD patients have impaired attentional set-shifting abilities, but not impaired task-set switching abilities [1].

Neurodegeneration in the meso-striatal dopaminergic system is a primary neuropathological feature of PD. A consensus regarding the relationship between the meso-striatal pathologies and the motor deficits that are observed in PD has been reached [11]. Similarly, a classic hypothesis suggests that cognitive inflexibility in PD arises from a disruption of meso-prefrontal and prefrontal-striatal circuits that is associated with dopaminergic insufficiency [1], [12], [13]; this hypothesis has been supported by several lines of evidence. First, executive dysfunction, including cognitive inflexibility, dominates the cognitive profiles of both PD patients and patients with prefrontal damage [1], [2], [6]. Second, levodopa administration improves WCST and other attentional set-shifting task performance in PD patients [4], [6], [8], [14], [15]. Lastly, functional magnetic resonance imaging (fMRI) studies have found evidence of a relationship between prefrontal dysfunction and poor performance on set-shifting tasks in PD patients [16], [17], [18], [19]. However, the results of recent studies have challenged the classic dopamine insufficiency hypothesis of cognitive inflexibility in PD patients. First, the administration of levodopa has been shown to have a task-specific cognitive benefit in PD patients: levodopa administration results in improved performance on the WCST, but it has no impact on the ID/ED task performance, which indicates that dopaminergic insufficiency may be associated with cognitive deficits other than attentional set-shifting [1]. Second, a recent study reported that patients with very early stages of PD, in whom neurodegeneration appears to be relatively confined to the dopaminergic systems, achieved performance scores on a task-set switching task that were within the normal range [9]. In agreement with these neuropsychological findings, which suggest that non-dopaminergic, extra-striatal pathologies to the set-shifting deficits that are observed in PD patients, recent structural neuroimaging studies have demonstrated that a degenerative process encroaches on the cerebral cortex and limbic structures in the early stages of the disease [20], [21], [22].

Research in cognitive neuroscience has shown that the prefrontal and the posterior parietal cortices work together in subserving both attentional set-shifting and attentional control in general [23], [24], [25], [26], [27]. Because these cortical regions can be affected in the early stages of PD [20], [21], [22], there is a possibility that parietal dysfunction plays a critical role in set-shifting deficits. To address this possibility, we should carefully avoid using tasks that require the involvement of ‘prefrontal-biased’ cognitive processes other than set-shifting, such as learning and working memory. In addition, current neuroimaging evidence for the neural correlates of set-shifting deficits in PD is primarily derived from activation studies: several fMRI studies have shown that PD patients have decreased levels of activation in the dorsolateral prefrontal cortex, the striatum and the parietal cortex when performing variants of the WCST [16], [18], [19]. Although fMRI has the advantage of enabling scientists to observe phasic brain activity while a subject performs a task, the brain regions in which phasic neural activity is decreased during task performance may differ from the brain regions in which at-rest neural activity is decreased [28]. Studies that investigate the correlation between lesions or at-rest-dysfunction and behavioral deficits are expected to provide supplementary evidence of the neural correlates of set-shifting deficits in PD. In this study, we used an 18F-*fluoro*-deoxy-glucose positron emission tomography (FDG-PET) technique and a compound letter paradigm to investigate the neural correlates of set-shifting deficits in PD patients. Compound letter paradigms have been used previously in neuroimaging studies of attentional control and attentional set-shifting [29], [30], [31], [32], [33] and in neuropsychological studies of PD [34], [35], [36]. As with other attentional set-shifting paradigms, such as the ID/ED task and the aforementioned paradigm that was used by Cools and colleagues, a compound letter paradigm has two distinct competing stimulus dimensions: the letter identity dimension (“?” or “?” in our task) and the global/local element dimension, between which cross-talk is present. The utility of this paradigm in the functional assessment of the fronto-parietal attentional network has been validated by several functional imaging studies [27], [29], [30], [31].

## Methods

All of the procedures that were used in this study were conducted in accordance with the guidelines of the Declaration of Helsinki and were approved by the Ethical Committee of the Tohoku University Graduate School of Medicine. All of the participants provided written informed consent after receiving a detailed explanation of the study.

### Subjects

Potential participants were identified at the movement disorder clinic at Tohoku University Hospital and were selected for participation on the basis of meeting all of the following criteria: (1) fulfillment of the diagnostic criteria for PD that were established by the UK PD Society Brain Bank [37]; (2) no history of other neurological or psychiatric diseases; (3) being between 55 and 75 years of age at the time of the study; (4) having an age of PD onset of more than 40 years old; (5) a Hoehn and Yahr stage of 1–3, (6) no magnetic resonance imaging (MRI) evidence of focal brain lesions, such as infarcts or tumors; (7) the absence of dementia as defined by the *Diagnostic and Statistical Manual of Mental Disorders*, Third Edition, Revised (DSM-IIIR), a Clinical Dementia Rating (CDR) stage of 0 or 0.5 [38] and a Mini-Mental State Examination (MMSE) [39] score ≥24; (8) no history of ocular disease and having a best-corrected Snellen visual acuity of 20/50 or better; and (9) the absence of diabetes mellitus. We provided detailed explanations of the study to all of the potential participants and/or their caregivers, and a total of 60 patients who provided written informed consent were enrolled in the study. Advertisements in the local community were used to recruit 30 healthy controls. Subjects with any history of neurological or psychiatric diseases, any cognitive impairment that was revealed during an interview and/or by an MMSE score of <24, or impaired visual acuity (a best-corrected Snellen acuity that was poorer than 20/50) were excluded from participation.

There were no significant differences between the PD (n = 60) and control (n = 30) groups in terms of age (66.2±5.8 vs. 66.0±5.3 years), sex (26 women/34 men vs. 17 women/13 men) or education (12.1±2.3 vs. 11.4±1.8 years) (Table 1). PD patients had significantly better visual acuity than the control participants (the median visual acuities of the two groups were 25/25 vs. 20/25, respectively). There was a trend toward having lower MMSE scores in the PD group compared with the control group (27.8±2.1 vs. 28.5±1.6). Of the 60 PD patients, 18 patients were not taking any dopaminergic agents, 10 were taking levodopa alone, and 32 were taking both levodopa and dopamine receptor agonists. Seven patients received anticholinergic medication, and two patients received selective serotonin reuptake inhibitors. The mean levodopa equivalent dose [40] of the patients was 658.83±825.5 mg/day. These and other demographic data are shown in **Table 1**.

**Table 1:** Demographic and clinical characteristics of patients with PD and control participants.

	PD (n = 60)	Controls (n = 30)	*p*-values
**Age** (years)	66.2±5.8	66.0±5.3	0.884
**Sex** (females/males)	26/34	17/13	0.232
**Level of education** (years)	12.1±2.3	11.4±1.8	0.151
**Visual acuity** (median)	20/20	20/25	0.027
**MMSE score**	27.8±2.1	28.5±1.6	0.074
**CDR stage** (0/0.5)	36/24		
**NPI depression score** (frequency × severity)	0.9±1.7		
**UPDRS-III**	19.9±7.4		
**Motor subtype** (tremor/akinetic-rigid)	38/22		
**Dominant side of motor symptoms** (L/R/B)	21/37/2		
**Disease duration** (years)	5.3±4.2		
**Levodopa equivalent dose** (mg/day)	658.83±825.5		

PD, Parkinson’s disease; MMSE, Mini Mental State Examination; CDR, Clinical Dementia Rating; NPI, Neuropsychiatric Inventory; UPDRS-III, Unified Parkinson’s Disease Rating Scale-motor score; L, left; R, right; B, bilateral.

### Psychophysical Tasks

**Figure 1:**
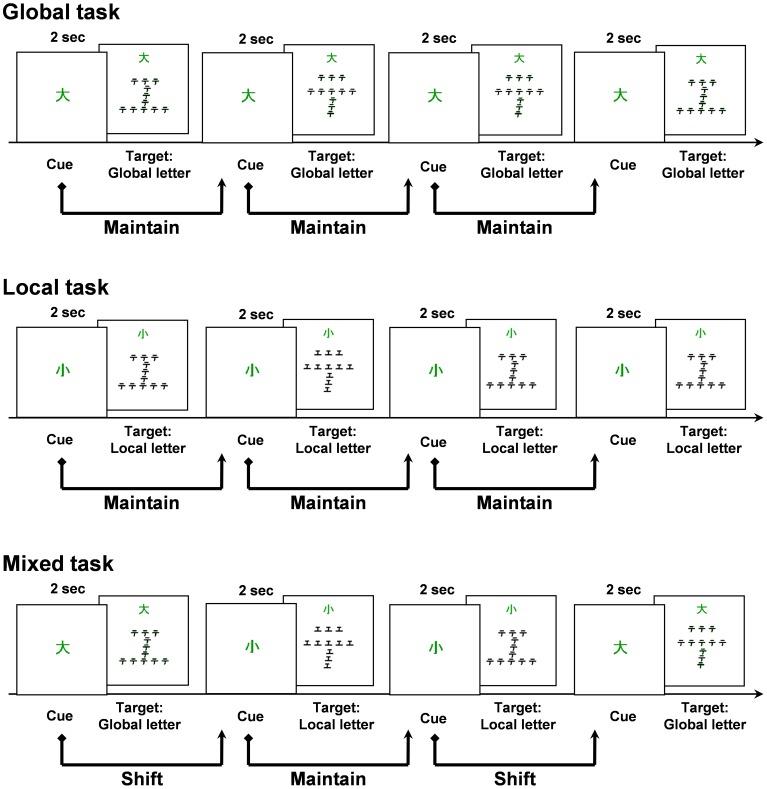
Schematic illustrations of the psychophysical tasks. In both the Global and Local tasks, compound letter stimuli appeared after a 2-second presentation of a visual cue that indicated whether the target was a global or local letter. The subjects were instructed to respond orally to the target component of each compound letter stimulus as quickly as possible. In these tasks, the subjects maintained their attention on a single component of the compound letters (either the local or global component of the stimuli), and they were not required to reorient their attention. However, in the Mixed task, the cue that indicated the target component of the compound letter changed from trial to trial in a pseudorandom manner. The task required that the subjects switch their attention on the basis of the cue that was presented to them on each trial.

Three different compound letter decision tasks were administered: the Global, Local, and Mixed tasks (**Figure 1**). Each subject completed five training trials and 24 test trials for each of the three tasks. The orders of the Global and Local tasks were counterbalanced between subjects. Visual stimuli were presented in the center of either a 17- or 15-inch liquid crystal display that was located at a distance of 70 cm from the subject. Two different compound letter stimuli were used throughout the tasks; one was a global “
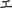
” that consisted of local “
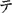
”s, and the other was a global “
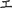
” that consisted of local “”s (“
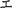
” and “
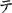
” are both Japanese Kana (phonographic characters). In each of the compound letter stimuli, a global letter (8.0 cm×8.0 cm, which subtended 6.5 degrees of visual angle) was composed of 11 small local letters (1.0 cm×1.0 cm, which subtended 0.8 degrees of visual angle). Subjects were instructed to read either the global letter or the local letter that was embedded in a compound letter stimulus aloud in accordance with the identity of a preceding cue as quickly as possible. Their oral responses were digitally recorded, and the reaction time (RT) of each trial was measured as the time between the onset of the visual stimulus and the onset of the oral response.

#### (a) Global and local tasks

In the Global task, compound letter stimuli appeared after a visual cue indicating that the target was a global letter (“?”, a Kanji (logogram) character meaning “large”) had been presented for 2 seconds (**Figure 1**). The subjects were then required to read the global letter in each compound letter stimulus aloud as quickly as possible. Compound letter stimuli remained visible on the screen until the subject responded. All of the procedures for the Local task were identical to those used in the Global task except that the initial visual cue indicated that the target was a local letter (“?”, meaning “small”), and the subjects were required to respond to the local letters. No task shifting occurred within either the Global task or the Local task, and the subjects focused their attention on the same component of the compound letter stimuli throughout each task.

#### (b) Mixed task

Prior to the presentation of each compound letter stimulus, a visual cue indicating “global” or “local” was presented for 2 seconds in a pseudorandom order. Thus, subjects had to switch their attention between the global and local components of the compound letter stimuli on the basis of the cue. The other procedures that were used were identical to those that were used in the Global and Local tasks.

#### (c) Large and Small tasks

We employed two additional tasks, the Large and Small tasks, to rule out the possibility that any observed psychophysical differences in global and local processing were confounded by differences in stimulus size. In the Large task, subjects were asked to read aloud large letters that subtended 6.5 degrees of visual angle (8.0 cm×8.0 cm) and that were presented after a 2-second presentation of a fixation cross. The procedure for the Small task was the same as the procedure that was used for the Large task except that the letter stimuli were small in size and subtended 0.8 degrees of visual angle (1.0 cm×1.0 cm).

#### (d) Shift cost

We used shift cost as a measure of attentional set-shifting ability. The shift cost was calculated according to the following formula: *Shift cost  =  (mean RT on the Mixed task) – {(mean RT on the Global task) + (mean RT on the Local task)}/2*.

#### (e) Statistics

The mean RTs and error rates in the psychophysical tasks were analyzed using two-way repeated-measures analyses of variance (ANOVAs) in which the group (PD or control) was used as a between-subjects factor and the task (Global, Local, and Mix) was used as a within-subjects factor. The Greenhouse-Geisser correction was applied when the data violated the assumption of sphericity. Details of the post hoc analyses are described in the Results section. A two-sample t-test was used to make a between-group comparison of the shift cost.

To identify the confounding factors in the regression analyses for the psychophysical measures and positron emission tomography (PET) data (brain-behavior analyses), we conducted analyses that sought to identify correlations between psychophysical task performance and other clinical data (i.e., MMSE, Neuropsychiatric Inventory (NPI) depression score [41], Unified Parkinson’s Disease Rating Scale-motor part (UPDRS-III) [42], and levodopa equivalent dose) in the PD group.

### Positron Emission Tomography (PET)

Each of the 60 PD patients underwent a PET scan within the 2 weeks that preceded or followed the clinical assessments. Prior to undergoing the PET scan, the patients had fasted, and their use of any dopaminergic medication(s) had been discontinued for at least 5 hours. Each patient received an injection of 185–218 MBq FDG, and scans were performed using a Siemens Biograph DUO scanner in 3D mode. After a 1-hour FDG-uptake period, each patient underwent a 20-minute scan during which the patient was awake, resting and wearing an eye mask. The in-plane and axial resolutions of the scan were 3.38 mm×3.38 mm, respectively. The data that were obtained were reconstructed to yield a 256×256 matrix with a pixel size of 1.33×1.33 mm and a slice thickness of 2.0 mm. The resultant images were analyzed using SPM5 (http://www.fil.ion.ucl.ac.uk/spm/software/spm5/). All of the images were normalized to the standard FDG template and were smoothed with a 10-mm full-width at half-maximum. Global normalization was performed using the “proportional scaling” and the relative threshold masking was set at 80% of the mean global value.

To identify the brain regions in which reductions in regional cerebral glucose metabolism (CMRglc) were associated with defective psychophysical performance, we conducted whole-brain voxel-based multiple regression analyses. The mean RT on each task or the shift cost was entered into each regression model as a variable of interest. We also included the age, sex, and clinical variables that were significantly correlated with psychophysical performance as nuisance variables. The height and extent thresholds were set at p<0.001 uncorrected and 100 voxels, respectively.

Subsequently, we performed region of interest (ROI)-based stepwise multiple regression analyses with the aim of exploring the relative contributions of the brain regions that had been identified in the whole-brain voxel-based analyses. Each regression model included the mean CMRglc values that were obtained within each of the ROIs as explanatory variables and either the mean RT on one of the psychophysical tasks or the shift cost as a dependent variable. The variables that were included in the regression models were selected on the basis of probabilities of F of ≤0.05 for inclusion and of ≥0.1 for removal. The ROIs were determined according to the following procedure: (1) the t-map images from the whole-brain voxel-based regression analyses for the Global, Local, and Mixed tasks (uncorrected p threshold <0.001 and size of 100 voxels or more) were transformed into binary images, after which (2) the overlapping areas from the three task conditions were extracted as ROIs.

Because we hypothesized that the psychophysical task performance impairments that we observed in PD patients resulted from brain dysfunction, we needed to verify that the brain regions that were identified in the regression analyses were hypometabolic in PD patients. To accomplish this, we compared the group CMRglc values from the 60 PD patients who participated in our study with the CMRglc values from another group of 14 healthy controls (age, 64.0±4.2 years; 7 men and 7 women; education level, 12.3±2.5 years; MMSE score, 29.1±1.3) who had not participated in the psychophysical tasks. The ages, sexes, and educational levels of these control subjects were comparable to those of the PD patients (age, p = 0.112; sex, p = 0.651; education, p = 0.753), and the same PET acquisition procedures that had been used for the PD patients were used to acquire metabolic data. Because of the referential purpose of the analysis, we employed a lenient height threshold (an uncorrected p threshold of <0.05), and we did not include any nuisance variables in the model (t-test).

## Results

### Psychophysical Tasks

#### 1. The effect of stimulus size

A two-way repeated-measures ANOVA in which task (Large and Small) was used as a within-subjects factor and group (PD and control) was used as a between-subjects factor revealed a trend toward a group effect (F = 3.82, p = 0.054). Neither the effect of task (F = 0.01, p = 0.929) nor the interaction between the two factors (F = 0.19, p = 0.662) was significant. These results suggest that the size of the stimulus had a negligible effect on performance in the compound letter tasks.

#### 2. The compound letter tasks

**Figure 2:**
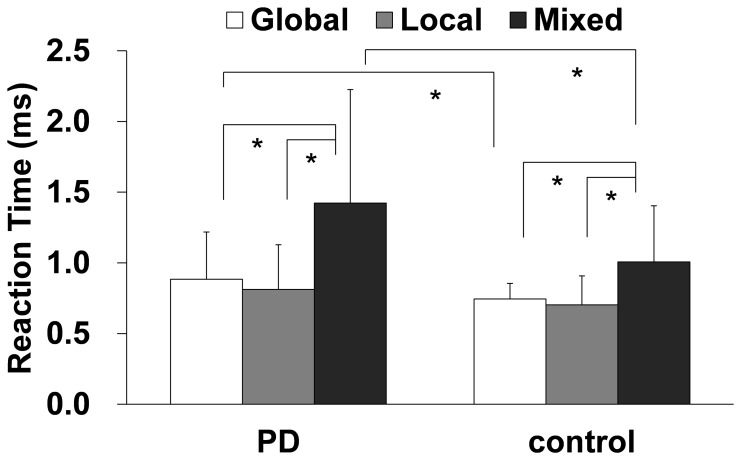
Mean RTs and error rates in the psychophysical tasks. Comparisons that were significantly different are indicated with a * (p<0.05/3). There was a significant simple interaction between group and the Global/Mixed task factor (F = 5.99, p = 0.016), and there was a trend toward an interaction between the group and the Local/Mixed task factor (F = 5.63, p = 0.020). PD, Parkinson’s disease.

A two-way repeated-measures ANOVA that used task (Global, Local, and Mixed) and group (PD and control) as factors revealed significant effects of both group (F = 7.06, p = 0.016) and task (F = 43.33, p = 0.001) and a significant interaction between the two factors (F = 5.00, p = 0.001) (**Figure 2**). The post hoc group comparisons for the three individual tasks (significance level p<0.05/3) showed that compared to the controls, the PD patients had significantly longer mean RTs in both the Global and Mixed tasks (Global, p = 0.004; Mixed, p = 0.001). There was also a trend toward longer mean RTs in the Local task in the PD group compared to the control group (p = 0.093). The between-task comparisons for each group (at a significance level of p<0.05/3) revealed that the mean RTs were significantly longer for the Mixed task than for either the Global or the Local task in both the PD and control groups (Global vs. Mixed, p = 0.001; Local vs. Mixed, p = 0.001 in both groups). No significant differences between the Global and Local tasks were identified in either group (p = 0.118 in the PD group, p = 0.260 in the control group). In addition, we found a significant interaction (significance level p<0.05/3) between the Mixed and Global tasks (F = 5.99, p = 0.016) and a trend between the Mixed and Local tasks (F = 5.63, p = 0.020). There was no significant interaction between the Global and Local tasks (F = 5.63, p = 0.209). In summary, the RTs for the Mixed task were disproportionately longer than for either the Global task or the Local task in the PD patients compared to control participants (**Figure 2**).

We found one outlier PD patient whose mean RT on the Global task was longer than the mean RT of all of the PD patients +3 SDs. The results of the analysis were unchanged after we removed this patient; a two-way ANOVA that used group (PD vs. controls) as a between-subjects factor and task as a within-subjects factor yielded significant effects of both group and task and a significant interaction between the two factors (task, F(1,129) = 55.02, p = 0.001; group, F(7,87) = 7.65, p = 0.007; interaction, F(1,129) = 5.03, p = 0.015).

#### 3. Shift cost

The shift cost in the PD group was significantly greater than in the control group (0.57±0.59 in PD; 0.28±0.34 in controls; t = 2.51, p = 0.014).

#### 4. Error rates

Because of the very low error rates on the psychophysical tasks (the error rates for the Global, Local, and Mixed tasks were 2.43±4.68, 1.11±3.42, and 6.32±8.35%, respectively, in the PD group and 1.11±2.43, 0.56±1.44, and 2.92±3.12%, respectively, in the control group), we used angular-transformed data in the statistical analyses. We performed a two-way repeated-measures ANOVA in which task (Global, Local, and Mixed task) and group (PD and controls) were factors, and we identified a significant main effect of task (F = 8.75, p = 0.001). We did not detect a significant effect of group (F = 2.33, p = 0.13), nor did we find a significant interaction between group and task (F = 0.57, p = 0.511). Post hoc comparisons revealed that the error rates for the Mixed task were greater than the error rates for the Local task in both PD patients and controls (p = 0.002 in PD, p = 0.006 in controls).

#### 5. Correlation between task performance and other clinical variables

We found that both the Mixed task RT and the shift costs were significantly correlated with the MMSE scores (r = −0.40, p = 0.001; r = −0.41, p = 0.001, respectively) and their UPDRS-III scores in the PD patients (r = 0.34, p = 0.007; r = 0.36, p = 0.005). A significant correlation between the Global task RT and the NPI depression score was also identified (r = 0.29, P = 0.023). There was no significant correlation between psychophysical task performance and levodopa equivalent dose.

### Positron Emission Tomography

**Figure 3:**
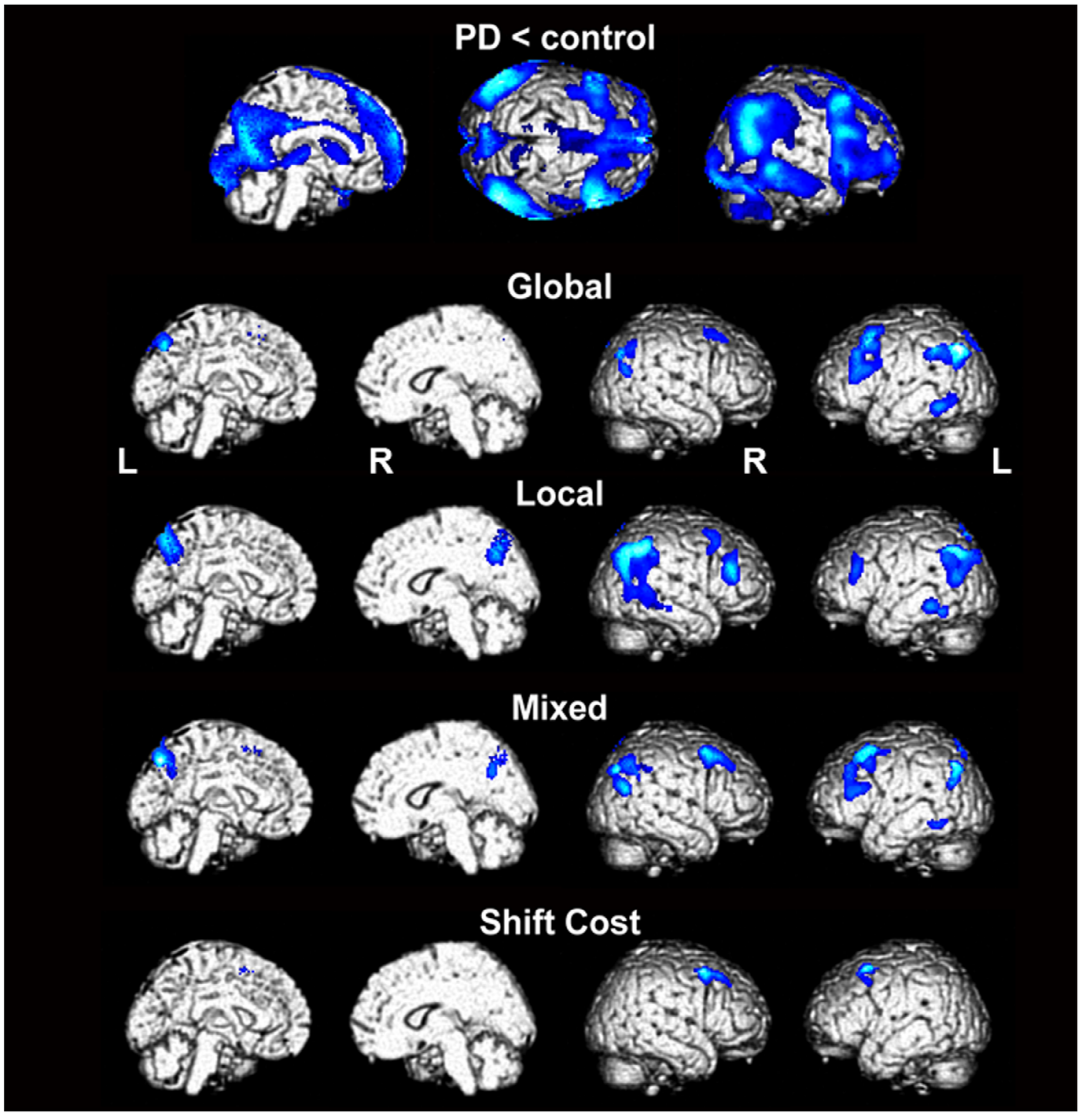
Results of the whole-brain voxel-based analyses. First row: The brain regions that exhibited regional cerebral glucose metabolic reductions in the 60 PD patients relative to 14 normal volunteers (p<0.05 uncorrected, extent threshold of 100 voxels). Second row and below: The brain regions in which the resting CMRglc was correlated with the RTs in the various psychophysical tasks (Global: second row, Local: third row, Mixed: fourth row) and the shift cost (fifth row) (*p*<0.001 uncorrected, extent threshold of 100 voxels). PD, Parkinson’s disease; R, right; L, left.

The results of the whole-brain voxel-based multiple regression analyses in which age, sex, MMSE score, and UPDRS-III were included as nuisance variables are shown in **Table 2** **and** **Figure 3**. For reference purposes, images that depict maps of sites at which there were reductions in CMRglc in the 60 PD patients relative to the 14 healthy controls are presented in **Figure 3** and in **Supplementary Figure S1**. Although the NPI depression scores were only correlated with the Mixed task RTs, previous studies have suggested that depression has a significant impact on cognitive function. We performed supplementary analyses in which the NPI depression score was included as a nuisance variable. The results of these analyses are shown in **Supplementary Figure S2**.

**Table 2:** Brain regions in which regional cerebral glucose metabolism was negatively correlated with psychophysical task response time.

Task	Regions	BA	MNI coordinates			Voxel-level		Cluster-level	
			x	y	z	t-value	Uncorrected*p-*value	Cluster size	Corrected*p-*value
Global	L inferior frontal gyrus	48	−46	22	22	4.79	<0.001	1308	<0.001
	L middle frontal gyrus	9	−44	14	44	4.69	<0.001		
	L middle frontal gyrus	45	−42	30	36	4.62	<0.001		
	L inferior temporal gyrus	37	−60	−48	−12	4.56	<0.001	308	0.116
	L superior parietal lobule	7	−12	−72	46	4.54	<0.001	1570	<0.001
	L middle occipital gyrus	7	−30	−68	40	4.44	<0.001		
	L supramarginal gyrus	40	−64	−40	36	4.41	<0.001		
	R angular gyrus	39	56	−64	46	4.3	<0.001	145	0.405
	R middle frontal gyrus	6	48	10	56	3.96	<0.001	146	0.402
	R middle frontal gyrus	9	42	22	52	3.67	<0.001		
	R middle temporal gyrus	39	50	−68	22	3.91	<0.001	297	0.126
	R middle occipital gyrus	7	34	−72	38	3.88	<0.001		
Local	R inferior frontal gyrus	45	56	32	22	5.23	<0.001	570	0.020
	L middle temporal gyrus	20	−66	−42	−8	5.09	<0.001	331	0.099
	L inferior temporal gyrus	37	−54	−52	−14	4.03	<0.001		
	L precuneus	7	−6	−72	56	4.86	<0.001	1022	0.001
	R precuneus	7	10	−60	34	4.06	<0.001		
	R middle occipital gyrus	39	48	−70	28	4.46	<0.001	2718	<0.001
	R inferior parietal lobule	39	60	−60	40	4.45	<0.001		
	R angular gyrus	39	48	−74	38	4.31	<0.001		
	L middle temporal gyrus	37	−50	−64	14	4.28	<0.001	1450	<0.001
	L angular gyrus	39	−56	−60	34	4.26	<0.001		
	L middle occipital gyrus	19	−34	−86	36	4.04	<0.001		
	L inferior frontal gyrus	45	−48	32	18	3.98	<0.001	275	0.150
	R middle frontal gyrus	9	50	14	42	3.9	<0.001	251	0.180
	R middle frontal gyrus	9	44	14	58	3.86	<0.001		
	R middle frontal gyrus	9	46	18	50	3.84	<0.001		
Mixed	R angular gyrus	39	54	−64	48	4.91	<0.001	840	0.004
	R middle temporal gyrus	39	48	−68	22	4.38	<0.001		
	R angular gyrus	19	44	−78	38	3.74	<0.001		
	R middle frontal gyrus	6	46	12	54	4.87	<0.001	559	0.021
	R middle frontal gyrus	9	38	34	42	3.82	<0.001		
	L middle occipital gyrus	7	−28	−68	40	4.65	<0.001	1272	<0.001
	L angular gyrus	39	−40	−66	28	4.42	<0.001		
	L precuneus	7	−8	−72	52	4.21	<0.001		
	L middle frontal gyrus	8	−26	10	54	4.46	<0.001	1232	<0.001
	L precentral gyrus	9	−44	12	44	4.39	<0.001		
	L middle frontal gyrus	9	−34	14	56	4.37	<0.001		
	L inferior temporal gyrus	37	−58	−54	−12	4.02	<0.001	112	0.521
Shift Cost	R middle frontal gyrus	6	48	10	52	4.27	<0.001	330	0.101
	R middle frontal gyrus	9	42	24	50	3.9	<0.001		
	R middle frontal gyrus	8	34	6	56	3.55	<0.001		
	L middle frontal gyrus	8	−24	10	54	4.24	<0.001	253	0.179
	L middle frontal gyrus	9	−36	12	56	3.92	<0.001		
	L precentral gyrus	9	−44	12	44	3.63	<0.001		

The CMRglc values in the bilateral frontal cortices were negatively correlated with the mean RTs when performing the Global task (**Table 2** **and** **Figure 3**). There were also significant negative correlations between the participants’ mean RTs when performing the Local task and their resting CMRglc values in the right frontal cortex, the bilateral temporo-parieto-occipital junctions (TPOs), the left posterior inferior temporal cortex, and the bilateral medial parietal cortices. In the Mixed task, the CMRglc values in the bilateral frontal cortices, bilateral TPOs, and left medial parietal cortex were negatively correlated with the mean RT. In addition, the shift cost was negatively correlated with the CMRglc values that were obtained from the bilateral frontal cortices. These results were generally unchanged when the NPI depression score was added to the regression model as a nuisance variable (**Supplementary Figure S2**).

**Figure 4:**
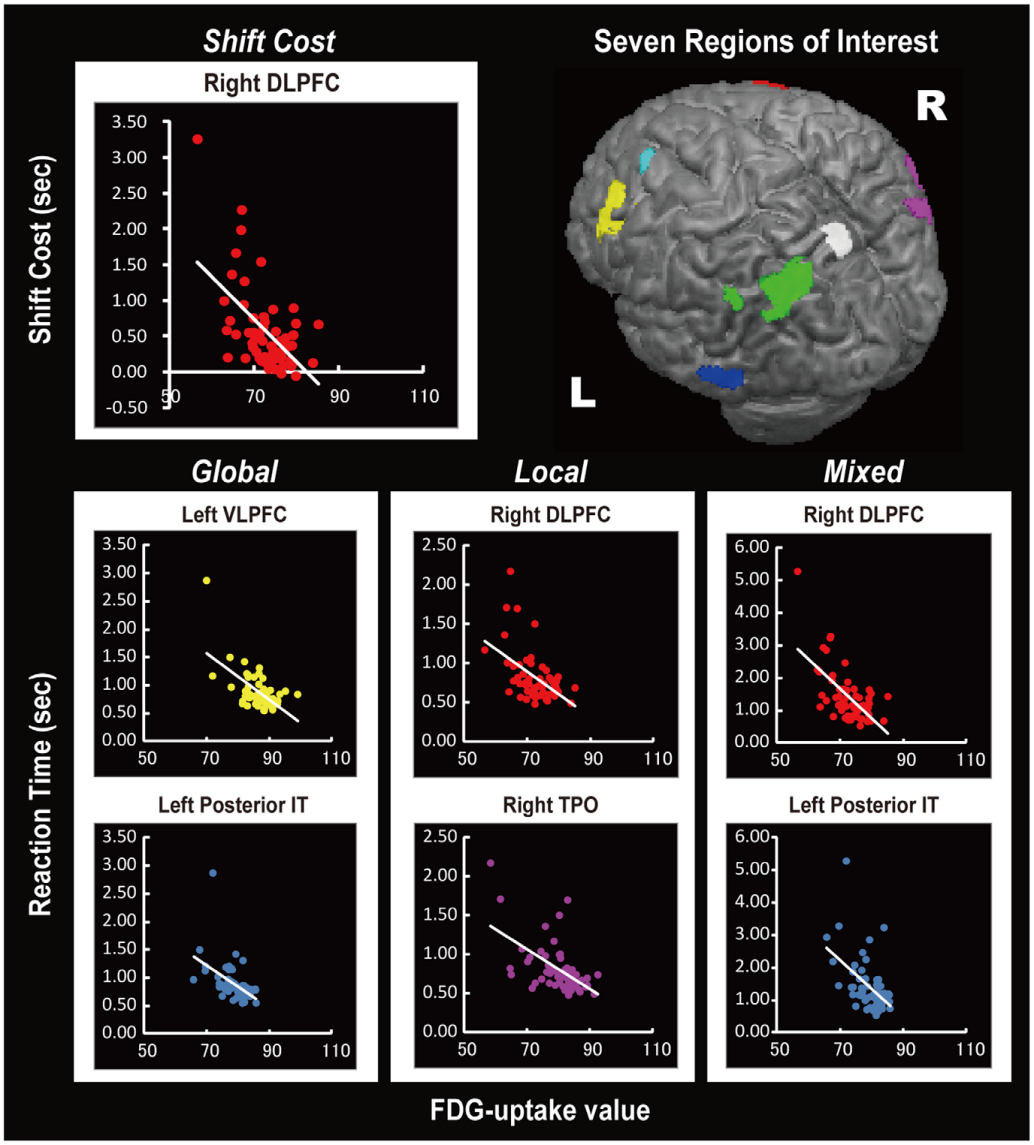
The results of the ROI-based stepwise multiple regression analyses. 7 ROIs are shown in different colors: right DLPFC  =  red, left DLPFC  =  cyan, left VLPFC  =  yellow, right TPO  =  purple, left TPO  =  green, medial parietal cortex  =  white, and left posterior IT  =  blue. The scatterplots illustrate the relationship between the psychophysical task performance scores and the FDG-uptake values in the ROIs. DLPFC, dorsolateral prefrontal cortex; VLPFC, ventrolateral prefrontal cortex; TPO, temporo-parieto-occipital junction; posterior IT, posterior inferior temporal cortex; FDG, ^18^F-fluorodeoxyglucose.

Subsequent stepwise multiple regression analyses were conducted using 7 ROIs; namely, the right and left dorsolateral prefrontal cortices (DLPFCs), the left ventrolateral prefrontal cortex (VLPFC), the left posterior inferior temporal cortex (posterior IT), the right and left TPOs, and the left medial parietal cortex. Reductions in CMRglc in the left VLPFC and left posterior IT were predictive of longer RTs on the Global task, whereas reductions in CMRglc in the right DLPFC and right TPO predicted longer RTs on the Local task. Hypometabolism in the right DLPFC and left posterior IT regions predicted longer RTs when performing the Mixed task. The shift cost was best predicted by hypometabolism in the right DLPFC (**Table 3** and **Figure 4**). When the NPI depression score was added to the regression model, reduced CMRglc values in the right DLPFC and the left posterior IT cortex predicted a larger shift cost (**Supplementary Table S1**). The results of the regression analysis in which the NPI depression score was added to the model were otherwise the same as those of the analyses in which the NPI depression score was not included in the model.

**Table 3:** Results of the ROI-based multiple regression analyses.

Task	Regions	Beta	Error	*t*-value for beta weight	*p*-value	R^2^
**Global**	Left ventrolateral prefrontal cortex	−0.476	0.008	−4.261	<0.001	0.405
	Left posterior inferior temporal cortex	−0.283	0.008	−2.535	0.014	
**Local**	Right dorsolateral prefrontal cortex	−0.296	0.007	−2.427	0.018	0.355
	Right temporo-parieto-occipital junction	−0.406	0.006	−3.330	0.002	
**Mixed**	Right dorsolateral prefrontal cortex	−0.511	0.015	−4.871	<0.001	0.446
	Left posterior inferior temporal cortex	−0.295	0.019	−2.815	0.007	
**Shift Cost**	Right dorsolateral prefrontal cortex	−0.562	0.011	−5.181	<0.001	0.305

## Discussion

### Attentional Set-shifting Deficit in PD

PD patients often have impaired performance on classic neuropsychological tests of ‘frontal-lobe’ functioning, such as the WCST and the ID/ED paradigms, which has led to the hypothesis that the set-shifting deficit that has been observed in PD patients arises from a disruption of the meso-prefrontal and prefrontal-striatal circuits [3], [4], [6]. However, the degree to which cognitive processes that are not involved in set-shifting, such as rule learning, concept formation and working memory, affect performance on these tasks is not clear [8]. Recent studies have made efforts to eliminate these confounding factors by using tasks that isolate set-shifting from other cognitive processes. For instance, in a series of studies by Cools, Kehagia and colleagues, subjects learned the associations of character types and background color cue or stimulus positions immediately before the test sessions [8], [9]. The subjects were then instructed to respond to either a digit or a letter that were presented side-by-side in accordance with the cues. Although their paradigm greatly reduces the working memory and concept formation loads in comparison to the WCST and the ID/ED task, the effects of cognitive processes other than set-shifting can be further reduced. The simultaneous presentation of cues and target stimuli in their task demands dual-task processing, and the maintenance of newly learned associations between cues (colors or positions) and targets (characters) requires working memory [29], [43]. We reduced the dual-task demands in our task by presenting the cues prior to the target stimuli, and the semantically explicit associations between the cues and the targets diminished the working memory load. A 2-second delay between cue onset and stimulus onset allowed the subjects to select a behavioral response prior to stimulus presentation. Consequently, the increase in shift cost that was observed in this study can be interpreted as a deficit in the post-selection attentional orienting mechanism in the presence of competing stimuli [1], [7], [8].

Before we can conclude that PD patients have attentional set-shifting deficits from the results of this study, we should address the possible confounding effects of bradykinesia and psychomotor slowing (bradyphrenia). Although an oral response was used in place of a button press to reduce the effect of motor deficits, the RTs of the PD patients were longer than the RTs of the controls on all of the psychophysical tasks. In addition to any residual motor deficit effects, psychomotor slowing may be associated with the general prolongation of RTs. However, neither the interactions between the Mixed task and the Global/Local tasks nor the increased shift cost in PD patients relative to control subjects are explicable in terms of such general effects, which indicates that there is an attentional set-shifting deficit in PD.

### Neural Correlates of an Attentional Set-shifting Deficit in PD

Recent evidence from cognitive neuroscience suggests that the prefrontal and parietal cortices cooperate in attentional set-shifting and, more broadly, attentional control [23], [24], [25], [26], [27]. It has been suggested that the prefrontal cortices are involved in task-specific (i.e., top-down) attentive processes, whereas the parietal cortices are considered to be engaged in stimulus-driven (i.e., bottom-up) attention [24], [44]. The long-standing hypothesis that the set-shifting deficit in PD arises from prefrontal dysfunction that is secondary to dopaminergic lesions in the midbrain has led to a relative neglect to consider the roles of the parietal cortices. Because neurodegeneration during the early stages of PD encroaches on not only the meso-striatal and meso-prefrontal dopaminergic systems but also on extensive cortical regions [20], [21], [22], we should consider the contributions of the parietal lesion along with the contribution of the meso-prefrontal and prefrontal-striatal lesions to the deficit. There have been several fMRI studies that directly addressed the neural correlates of the set-shifting deficit in PD. These fMRI studies demonstrated that the activation levels of the prefrontal cortices, striata, and parietal cortices of PD patients differed from those of normal subjects when performing the WCST [16], [18]. However, the use of the WCST precludes a straightforward interpretation of the results of these studies; the altered brain activation observed in these studies may reflect deficits in cognitive processes other than set-shifting, such as concept formation or working memory. We noted the same problem in a study by Marie and colleagues that investigated the correlation between at-rest striatal dopamine status, which was measured using 11C-S-Nomifensine PET, and an object alternation task [45]. Another line of evidence for the involvement of the meso-prefrontal and prefrontal-striatal pathologies in PD-associated set-shifting deficits has arisen from psychopharmacological studies. Several studies have demonstrated that dopaminergic drugs have ameliorating effects on the performances of PD patients on a variety of set-shifting tasks [3], [4], [14], [15]. The results of these studies have been interpreted as evidence in favor of the hypothesis that the disruption of prefrontal-striatal neural circuits plays a pivotal role in the attentional set-shifting deficits that are associated with PD. However, the dopaminergic modulation of cognition is not exclusively mediated by prefrontal-striatal circuits; the modulation of cognition is also mediated by direct action on dopamine receptors in the cerebral cortex. Similarly, a recent fMRI study revealed that levodopa administration in of PD patients who are performing the WCST results in changes in the activation of the motor circuits of the premotor cortex and putamen but does not alter the activation of the cognitive circuits of the prefrontal cortex, caudate and parietal cortex [46]. In this study, we used two methods to investigate the neural correlates of the attentional set-shifting deficit that has been observed in PD patients: a compound letter paradigm in which the reliance upon non-set-shifting cognitive processes, which are confounding factors in many studies, is greatly reduced; and FDG-PET, a neuroimaging method that is sensitive to at-rest neural dysfunction. Our results provide clear evidence for a relationship between prefrontal dysfunction and an attentional set-shifting deficit in PD; evidence of a similar relationship between parietal dysfunction and the attentional set-shifting deficit was not observed. These results are supported by a recent psychophysical study that demonstrated that PD patients have attenuated top-down attentional control and enhanced stimulus-driven attentional processing; the former depends primarily on prefrontal function, and the latter depends primarily on the parietal cortices [47].

The metabolic changes that were observed in the posterior IT cortex and the VLPFC were correlated with the patients’ performances on the Global task. The posterior IT, which is situated in the ventral visual pathway, is a cortical region that is devoted to the processing of complex visual forms, such as objects, faces, and letters [48], [49]. Because the Global task requires the assembly of local parts into a single global form, the visual form-processing deficit that is associated with dysfunction in this brain region may have resulted in impaired performances of the PD patients on the Global task. A previous study also found evidence of a relationship between the visual form-processing deficit and posterior IT hypometabolism in early PD [50]. The VLPFC is anatomically interconnected with the temporal cortices via the uncinate fasciculus. This region reportedly participates in the encoding, retrieval, and selection of the information that is represented in the ventral visual pathway [51], [52]. In addition to having roles in memory, previous studies have suggested that the VLPFC contributes to executive attentional control. In an fMRI study by Hampshire and Owen, an association between VLPFC activation and the extradimensional shifting that was required in a modified ID/ED task was observed [53]. However, the role of the VLPFC in attentional set-shifting itself was obscured because their task used overlapping pictures of faces and houses in place of the abstract geometric figures that were used in the original ID/ED task. Thus, the VLPFC activation that they observed may have been related to semantic categorical shifting or, more broadly, to the manipulation of semantic categorical information, such as identifying faces and houses. In addition, the right VLPFC has been in implicated in response inhibition in a number of human and animal studies [7], [54]. In this study, diminished Global task performance was more clearly associated with a reduction in CMRglc in the left VLPFC than in the right VLPFC. This left hemispheric dominance may be attributable to the demand for language processing in the compound letter task. The rapid matching of the relatively ambiguous forms of the global letters to letter forms that are stored in the long-term memory may be related to the involvement of the left VLPFC.

Hypometabolism in the right DLPFC predicted both a longer RT on both the Local and Mixed tasks and an increased shift cost. This region of the DLPFC includes the intersection of the superior frontal and precentral sulci, which is called the putative human frontal eye field (FEF) [55]. The human FEF and the inferior parietal cortex form the dorsal fronto-parietal network, which is involved in the top-down control of attention that is driven by cognitive factors, including a current goal, prior knowledge, or expectation [24], [44]. The results of the Mixed task and shift cost were unsurprising because the task was explicitly designed to measure attentional control. However, we did not expect to observe a correlation between hypometabolism in this region and a prolonged mean RT in the Local task. Coupled with the longer mean RT that was observed in the Local task relative to the Global task, the involvement of another component of the dorsal fronto-parietal network, the TPO, suggests that the Local task demanded attentional control abilities [24], [29]. Successful performance in the Local task may require the recruitment of the dorsal fronto-parietal network to reorient attention and to focus it on small, local areas of continuously changing visual stimuli.

### Limitations

This investigation did not address the question of whether the attentional set-shifting deficit that was observed in PD patients is associated with lesions in either the cerebral cortex or subcortical structures, such as the striatum and the dopaminergic nuclei of the midbrain. The hypometabolism in the DLPFC that was observed in our study can arise from either prefrontal lesions or the disruption of prefrontal-subcortical circuits [56], [57]. Multiple neuroimaging techniques, such as dopaminergic PET and volumetric MRI, must be used to differentiate between the contributions of cortical and subcortical pathologies to cognitive dysfunction in patients with early stages of PD.

Previous studies have demonstrated that dopaminergic medication status has a significant impact on brain glucose metabolism. In particular, the CMRglc values in subcortical structures such as the striatum and the thalamus were increased by the administration of dopaminergic medication [58], [59]. Although we withheld dopaminergic medication for the 5 hours that immediately preceded the PET scan of each patient, this wash-out time is shorter in duration than the wash-out time that has been used in previous studies. It is possible that we failed to detect striatal metabolic abnormalities as a result of the effects of residual dopaminergic agents.

Compound letter paradigms have been used to investigate global and local processing in object perception [29], [30], [33], [43]. Although a number of previous studies have demonstrated a preference for global processing, the RTs for the Local task tended to be shorter than those for the Global task in our study. This discrepancy may be due to differences between our study and others in terms of the sizes of the stimuli, the number of local components that constitute a global object and the salience of the visual stimuli [32]. Unfortunately, our study did not address these issues. In addition, it has been reported that the laterality of brain pathology has an impact on compound letter task performance. For example, Schenden and colleagues reported that PD patients with left-dominant motor symptoms (which are indicative of right-dominant brain pathology) had more substantial impairments in global processing, whereas patients with right-dominant motor symptoms had more substantial impairments in local processing [60]. Although we failed to reproduce their findings in our supplementary analysis (see **Supplementary Experiments S1; Supplementary Tables S2 and S3; and Supplementary Figure S3**), this inconsistency may also arise from differences between their experiment and ours in both the subject populations and the physical features of the visual stimuli that were used.

## Supporting Information

Figure S1 The brain regions exhibiting a regional cerebral glucose metabolic (CMRglc) increase (red) and decrease (blue) in the 60 PD patients relative to the 14 normal volunteers (*p*<0.05 uncorrected, extent threshold of 100 voxels). We found no brain regions in that CMRglc was positively correlated with reaction times.
(TIF)

Figure S2 Results of the whole-brain voxel-wise analyses in that the NPI depression score was covaried out.
(TIF)

Figure S3 Results of post hoc pairwise comparisons in a 2-way ANOVA. *, p<0.05; **, p<0.01; ^†^, p<0.1. Tukey’s correction for multiple comparisons.
(TIF)

Table S1 Results of the ROI-based multiple regression analyses in that the NPI depression score was covaried out.
(DOCX)

Table S2 Demographic data of the patients with left- and right-lateralized motor symptoms.
(DOCX)

Table S3 Demographic data of the patients with tremor-type and non-tremor-type PD.
(DOCX)

Experiments S1 Impacts of the lateralization of motor symptoms and the motor subtypes on attentional set-shifting. Previous studies suggested that the lateralization of motor symptoms (lateralization of pathology) and the motor subtypes (tremor type or akinetic-rigid type) have an impact on cognitive performance in Parkinson’s disease (PD). Here, we address the impacts of these factors on the performance of our task.
(DOCX)
