# Supplementary figures and images for: Attentional Set-Shifting Deficit in Parkinson’s Disease Is Associated with Prefrontal Dysfunction: An FDG-PET Study
Source: PLoS One. 2012 Jun 7;7(6):e38498. doi: 10.1371/journal.pone.0038498 (PMC3369918; doi:10.1371/journal.pone.0038498)

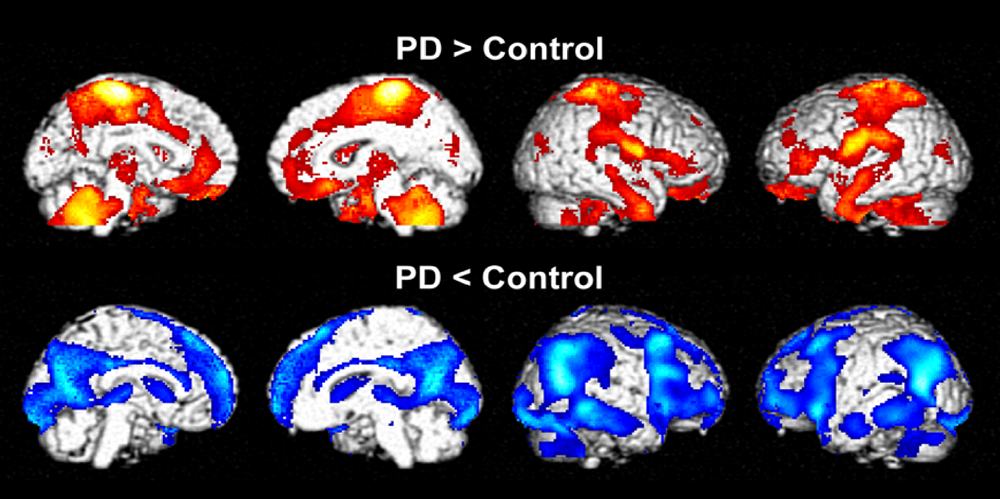

Supplement: Figure S1 — The brain regions exhibiting a regional cerebral glucose metabolic (CMRglc) increase (red) and decrease (blue) in the 60 PD patients relative to the 14 normal volunteers (p<0.05 uncorrected, extent threshold of 100 voxels). We found no brain regions in that CMRglc was positively correlated with reaction times. (TIF) [file pone.0038498.s001.tif]

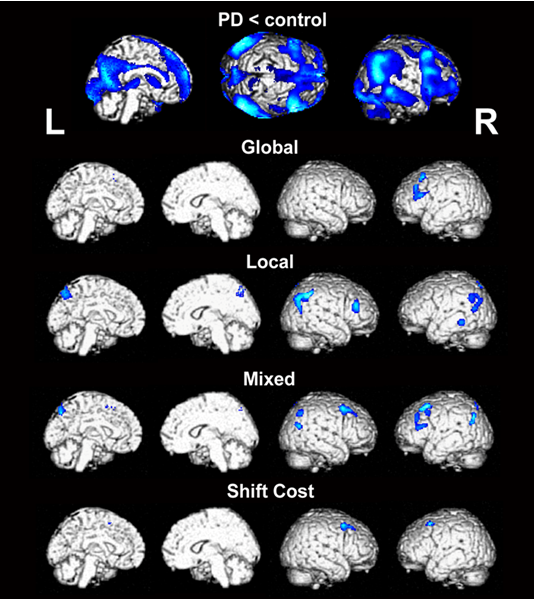

Supplement: Figure S2 — Results of the whole-brain voxel-wise analyses in that the NPI depression score was covaried out. (TIF) [file pone.0038498.s002.tif]

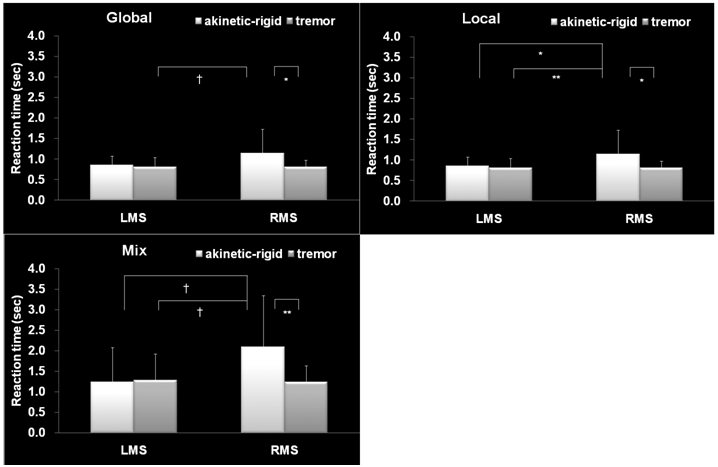

Supplement: Figure S3 — Results of post hoc pairwise comparisons in a 2-way ANOVA. *, p<0.05; **, p<0.01; †, p<0.1. Tukey’s correction for multiple comparisons. (TIF) [file pone.0038498.s003.tif]
