# Supplementary material for: Attentional Set-Shifting Deficit in Parkinson’s Disease Is Associated with Prefrontal Dysfunction: An FDG-PET Study
Source: PLoS One. 2012 Jun 7;7(6):e38498. doi: 10.1371/journal.pone.0038498 (PMC3369918; doi:10.1371/journal.pone.0038498)
Supplement: Table S1 — Results of the ROI-based multiple regression analyses in that the NPI depression score was covaried out. (DOCX) [file pone.0038498.s004.docx]

| **Supplementary table 1.** Results of the ROI-based multiple regression analyses in that the NPI depression score was covaried out. | | | | | | |
| --- | --- | --- | --- | --- | --- | --- |
| **Dependent variables** | **Region** | **Beta** | **Error** | ***t*-value for beta weight** | ***p*-value** | **R^2^** |
| Global | Left ventrolateral prefontal regions | -0.462 | 0.008 | -3.999 | <0.001 | 0.397 |
|  | Left posterior inferior temporal region | -0.271 | 0.009 | -2.372 | 0.021 |  |
|  | NPI depression score | 0.060 | 0.021 | 0.551 | 0584 |  |
| Local | Right dorsolateral prefrontal regions | -0.311 | 0.007 | -2.494 | 0.016 | 0.348 |
|  | Right temporo-parieto-occipital junction | -0.426 | 0.006 | -3.373 | 0.001 |  |
|  | NPI depression score | -0.075 | 0.021 | -0.654 | 0.516 |  |
| Mix | Right dorsolateral prefrontal regions | -0.546 | 0.016 | -5.054 | <0.001 | 0.452 |
|  | Left posterior inferior temporal region | -0.322 | 0.019 | -3.021 | 0.004 |  |
|  | NPI depression score | -0.131 | 0.049 | -1.256 | 0.214 |  |
| Shift Cost | Right dorsolateral prefrontal regions | -0.532 | 0.012 | -4.508 | <0.001 | 0.346 |
|  | Left posterior inferior temporal region | -0.240 | 0.015 | -2.062 | 0.044 |  |
|  | NPI depression score | -0.180 | 0.039 | -1.579 | 0.120 |  |
