# Supplementary material for: Attentional Set-Shifting Deficit in Parkinson’s Disease Is Associated with Prefrontal Dysfunction: An FDG-PET Study
Source: PLoS One. 2012 Jun 7;7(6):e38498. doi: 10.1371/journal.pone.0038498 (PMC3369918; doi:10.1371/journal.pone.0038498)
Supplement: Table S2 — Demographic data of the patients with left- and right-lateralized motor symptoms. (DOCX) [file pone.0038498.s005.docx]

| **Supplementary table 2.** Demographic data of the patients with left- and right-lateralized motor symptoms | | | |
| --- | --- | --- | --- |
|  | **LMS (n = 21)** | **RMS (n = 37)** | ***p*-values** |
| **Age**, years | 65.7 ± 5.2 | 66.1 ± 6.0 | 0.791 |
| **Sex** (female/male) | 10/11 | 22/15 | 0.384 |
| **Visual acuity** (median) | 50/50 | 50/50 |  |
| **MMSE score** | 28.0 ± 1.9 | 27.6 ± 2.2 | 0.570 |
| **NPI depression score** (frequency × severity) | 0.8 ± 1.5 | 1.0 ± 1.9 | 0.583 |
| **UPDRS-III score** | 17.7 ± 6.9 | 20.9 ± 7.5 | 0.106 |
| **Initial symptoms** (tremor/akinetic-rigid) | 13/8 | 25/12 | 0.190 |
| **Disease duration**, years | 5.2 ± 4.5 | 5.3 ± 4.1 | 0.960 |
| **Levodopa equivalent dose** (mg/day) | 461.5 ± 540.9 | 707.8 ± 865.3 | 0.244 |
| LMS, patients with left-lateralized motor symptoms; RMS, patients with right-lateralized motor symptoms; MMSE, Mini Mental State Examination; NPI, Neuropsychiatric Inventory; UPDRS-III , Unified Parkinson’s Disease Rating Scale motor score. | | | |
