# Supplementary material for: Attentional Set-Shifting Deficit in Parkinson’s Disease Is Associated with Prefrontal Dysfunction: An FDG-PET Study
Source: PLoS One. 2012 Jun 7;7(6):e38498. doi: 10.1371/journal.pone.0038498 (PMC3369918; doi:10.1371/journal.pone.0038498)
Supplement: Table S3 — Demographic data of the patients with tremor-type and non-tremor-type PD. (DOCX) [file pone.0038498.s006.docx]

| **Supplementary table 3.** Demographic data of the patients with tremor-type and non-tremor-type PD | | | |
| --- | --- | --- | --- |
|  | **Tremor (n = 38)** | **Akinetic-rigid (n = 20)** | ***p*-values** |
| **Age**, years | 66.4 ± 5.7 | 65.1 ± 5.8 | 0.426 |
| **Sex** (female/male) | 20/18 | 12/8 | 0.288 |
| **Visual acuity** (median) | 50/50 | 50/50 |  |
| **MMSE score** | 27.8 ± 2.1 | 27.6 ± 2.2 | 0.714 |
| **NPI depression score** (frequency × severity) | 0.8 ± 2.0 | 1.1 ± 1.3 | 0.597 |
| **UPDRS-III score** | 19.9 ± 7.6 | 19.4 ± 7.3 | 0.792 |
| **Side of initial motor symptoms** (left/right) | 13/25 | 8/12 | 0.190 |
| **Disease duration**, years | 5.5 ± 4.5 | 4.8 ± 3.7 | 0.538 |
| **Levodopa equivalent dose** (mg/day) | 584.5 ± 754.3 | 683.4 ± 809.1 | 0.645 |
| MMSE, Mini Mental State Examination; NPI, Neuropsychiatric Inventory; UPDRS-III, Unified Parkinson’s Disease Rating Scale motor score. | | | |

**Supplementary figure 3.** Results of post hoc pairwise comparisons in a 2-way ANOVA.


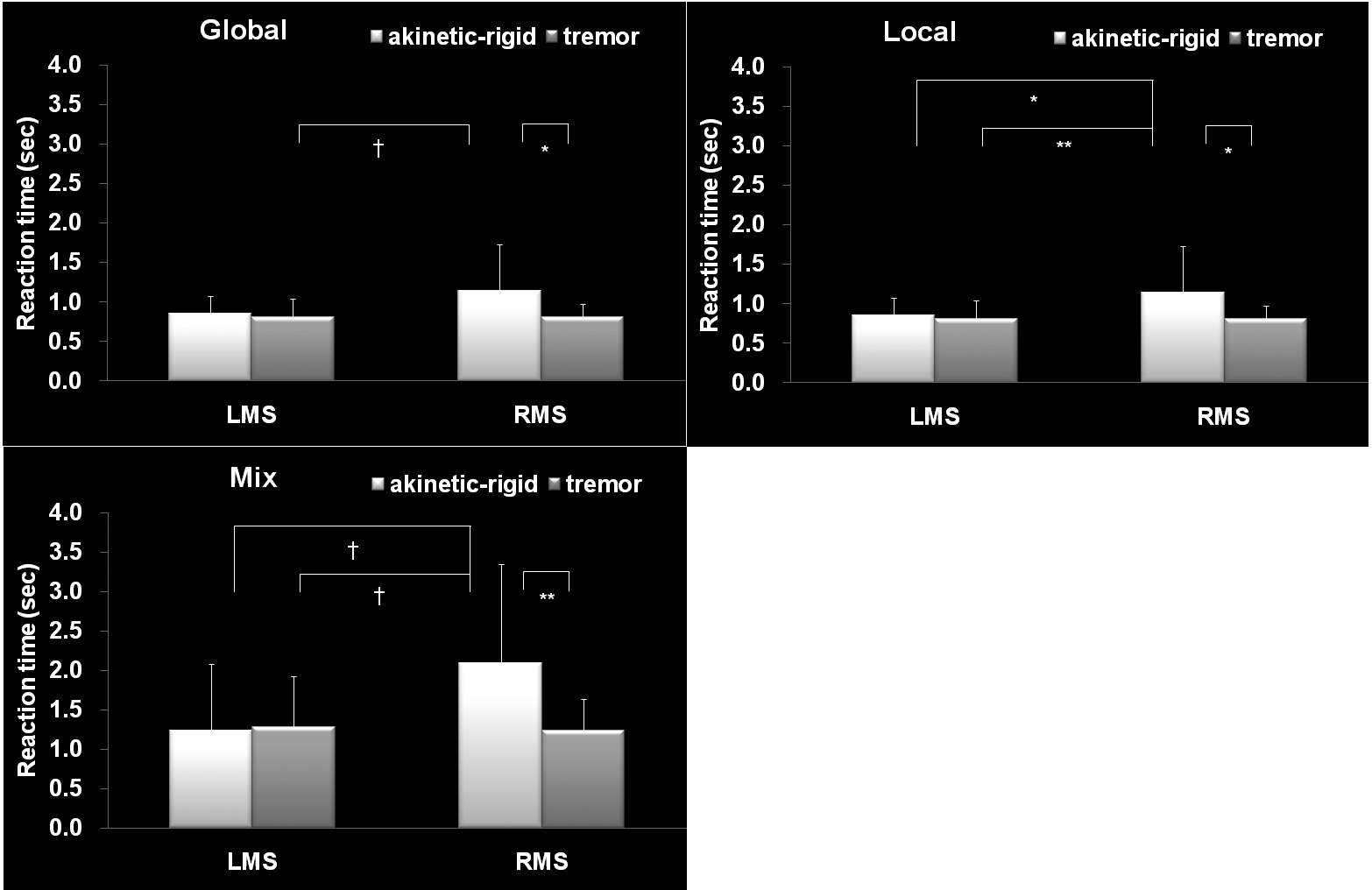


*, p < 0.05; **, p < 0.01; † , p < 0.1. Tukey’s correction for multiple comparisons.

LMS, patients with left-lateralized motor symptoms; RMS, patients with right-lateralized motor symptoms.
