# Supplementary material for: Attentional Set-Shifting Deficit in Parkinson’s Disease Is Associated with Prefrontal Dysfunction: An FDG-PET Study
Source: PLoS One. 2012 Jun 7;7(6):e38498. doi: 10.1371/journal.pone.0038498 (PMC3369918; doi:10.1371/journal.pone.0038498)
Supplement: Experiments S1 — Impacts of the lateralization of motor symptoms and the motor subtypes on attentional set-shifting. Previous studies suggested that the lateralization of motor symptoms (lateralization of pathology) and the motor subtypes (tremor type or akinetic-rigid type) have an impact on cognitive performance in Parkinson’s disease (PD). Here, we address the impacts of these factors on the performance of our task. (DOCX) [file pone.0038498.s007.docx]

**SUPPLEMENTARY EXPERIMENTS**

**Impacts of the lateralization of motor symptoms and the motor subtypes on attentional set-shifting**

Previous studies suggested that the lateralization of motor symptoms (lateralization of pathology) and the motor subtypes (tremor type or akinetic-rigid type) have an impact on cognitive performance in Parkinson’s disease (PD). Here, we address the impacts of these factors on the performance of our task.

**1. Methods.**

Of the 60 patients with PD, 2 patients were excluded from the analyses because of symmetrical motor symptoms. Accordingly, we analyzed 58 PD patients with lateralized motor symptoms. Demographic data are shown in **Supplementary tables 2 and 3**. First, we conducted a 3-way analysis of variance (ANOVA) with factors of tasks (Global, Local, or Mixed), motor laterality (left or right), and motor subtype (tremor or akinetic-rigid). An analysis of covariance (ANCOVA) was also performed with NPI depression score, UPDRS-III, and MMSE as nuisance variables. Next, we performed a 2-way ANOVA with factors of laterality and subtype in each of the Global, Local, and Mixed tasks. A 2-way ANCOVA was also performed with NPI depression score, UPDRS-III, and MMSE as nuisance variables.

**2. Results.**

**2.1.** **Three-way ANOVA and ANCOVA**

The main effects of task and motor subtype were significant in both ANOVA {task, F(1, 81) = 42.79, p < 0.001; subtype, F(1, 54) = 4.89, p = 0.031} and ANCOVA {task, F(2, 80) = 7.69, p = 0.002; subtype, F(1, 51) = 5.22, p = 0.027}. There was a significant main effect of laterality in ANOVA but not in ANCOVA {F(1, 54) = 5.29, p = 0.025 in ANOVA; F(1, 51) = 2.76, p = 0.103 in ANCOVA}.

A significant simple interaction between laterality and subtype was found in ANOVA {F(1, 54) = 4.36, p = 0.041} but not in ANCOVA {F(1, 51) = 2.363, p = 0.130}. Simple interactions between task and laterality and between task and motor subtype were not significant in either ANOVA or ANCOVA {task × laterality, F(1, 81) = 1.42, p = 0.236 in ANOVA, F(2, 80) = 1.301, p = 0.273 in ANCOVA; task × subtype, F(1, 81) = 1.47, p = 0.236 in ANOVA, F(2, 80) = 1.59, p = 0.214 in ANCOVA}.

**2.2. Two-way ANOVA and ANCOVA**

2.2.1. The Global task

The main effect of motor subtype was significant in both ANOVA and ANCOVA {F(1, 54) = 4.45, p = 0.040; F(1, 51) = 4.31, p = 0.043}. The main effect of laterality {F(1, 54) = 2.54, p = 0.117 in ANOVA; F(1, 51) = 1.05, p = 0.310 in ANCOVA} and the interaction between laterality and subtype {F(1, 54) = 2.57, p = 0.115 in ANOVA; F(1, 51) = 1.36, p = 0.248 in ANCOVA} were not significant. The results of post hoc pairwise comparisons among the 4 groups (LMS/tremor, LMS/akinetic-rigid, RMS/tremor, and RMS/akinetic-rigid) are shown in **Supplementary figure 3**.

2.2.2. The Local task

ANOVAs revealed significant main effects of laterality and motor subtype {laterality, F(1, 54) = 8.36, p = 0.006; subtype, F(1, 54) = 3.75, p = 0.058}, whereas ANCOVAs showed a significant trend of the main effect of laterality and a significant trend of motor subtype {laterality, F(1, 51) = 5.98, p = 0.018; subtype, F(1, 51) = 3.42, p = 0.070}. There was no significant interaction between the two factors {F(1, 54) = 2.00, p = 0.163 in ANOVA; F(1, 51) = 1.12, p = 0.296 in ANCOVA}. The results of post hoc pairwise comparisons are shown in **Supplementary figure 3**.

2.2.3. The Mixed task

There was a significant trend of the main effect of motor subtype in both ANOVA and ANCOVA {F(1, 54) = 3.44, p = 0.069; F(1, 51) = 3.77, p = 0.058}. A significant trend of the main effect of laterality was found in ANOVA but not in ANCOVA {laterality, F(1, 54) = 3.39, p = 0.071 in ANOVA; F(1, 51) = 1.35, p = 0.251 in ANCOVA}. There was a significant interaction between the two factors in ANOVA but not in ANCOVA {F(1, 54) = 4.31, p = 0.043 in ANOVA; F(1, 51) = 2.194, p = 0.145 in ANCOVA}. The results of post hoc pairwise comparisons are shown in **Supplementary figure 3**.

**3. Discussion**

We found that patients with right-lateralized motor symptoms (RMS) performed worse than those with left-lateralized motor symptoms (LMS) on the Local and Mixed tasks. Previous studies reported that patients with RMS (left-lateralized brain pathology) were more impaired on verbal tasks than were those with LMS. Opposite laterality effects were observed on visuospatial tasks (Taylor et al. 1986; Starkstein et al. 1987). Poorer performance on compound letter tasks in patients with RMS may be associated with their impairment in language or orthographic processing. Schendan and colleagues examined laterality effects on performance in compound letter paradigms that were similar to those used in our study (Schendan et al. 2009). Unlike our results, the authors found greater impairment of global processing in PD patients with LMS and greater impairment of local processing in patients with RMS. The inconsistency between our findings and those of previous studies may arise from confounding factors when attempting to compare patients with LMS to those with RMS, such as differences in the severity of motor and cognitive symptoms, motor subtype, and medication. Indeed, in our study, laterality effects disappeared when scores on the MMSE, NPI-depression, and UPDRS-III were covaried out. A recent study demonstrated an effect of the interaction between motor laterality and medication on executive function in PD (Tomer et al. 2007).

The deteriorating effects of the PD akinetic-rigid subtype on cognitive function have been demonstrated repeatedly (Emre 2003; Lewis et al. 2005; Emre et al. 2007; Williams-Gray et al. 2007). Consistent with these findings, we found that patients with the akinetic-rigid subtype of PD were more impaired on all of the compound letter tasks than were those with the tremor subtype . A recent clinico-pathological study demonstrated a relationship between the akinetic-rigid phenotype and neocortical pathology (Selikhova et al. 2009). Cortical or non-dopaminergic pathology may play a significant role in impairment of attentional control ability, which was required for our compound letter task.

**4. References**

Emre M. 2003. Dementia associated with Parkinson's disease. Lancet Neurol. 2: 229-237.

Emre M, Aarsland D, Brown R, Burn DJ, Duyckaerts C, Mizuno Y, Broe GA, Cummings J, Dickson DW, Gauthier S, Goldman J, Goetz C, Korczyn A, Lees A, Levy R, Litvan I, McKeith I, Olanow W, Poewe W, Quinn N, Sampaio C, Tolosa E, Dubois B. 2007. Clinical diagnostic criteria for dementia associated with Parkinson's disease. Mov Disord. 22: 1689-1707; quiz 1837.

Lewis SJ, Foltynie T, Blackwell AD, Robbins TW, Owen AM, Barker RA. 2005. Heterogeneity of Parkinson's disease in the early clinical stages using a data driven approach. J Neurol Neurosurg Psychiatry. 76: 343-348.

Schendan HE, Amick MM, Cronin-Golomb A. 2009. Role of a lateralized parietal-basal ganglia circuit in hierarchical pattern perception: evidence from Parkinson's disease. Behav Neurosci. 123: 125-136.

Selikhova M, Williams DR, Kempster PA, Holton JL, Revesz T, Lees AJ. 2009. A clinico-pathological study of subtypes in Parkinson's disease. Brain. 132: 2947-2957.

Starkstein S, Leiguarda R, Gershanik O, Berthier M. 1987. Neuropsychological disturbances in hemiparkinson's disease. Neurology. 37: 1762-1764.

Taylor AE, Saint-Cyr JA, Lang AE. 1986. Frontal lobe dysfunction in Parkinson's disease. The cortical focus of neostriatal outflow. Brain. 109 ( Pt 5): 845-883.

Tomer R, Aharon-Peretz J, Tsitrinbaum Z. 2007. Dopamine asymmetry interacts with medication to affect cognition in Parkinson's disease. Neuropsychologia. 45: 357-367.

Williams-Gray CH, Foltynie T, Brayne CE, Robbins TW, Barker RA. 2007. Evolution of cognitive dysfunction in an incident Parkinson's disease cohort. Brain. 130: 1787-1798.
